# Shaping individual competitiveness: The role of discipline, parental expectations, and participation in extracurricular activities

**DOI:** 10.1016/j.heliyon.2024.e31042

**Published:** 2024-05-11

**Authors:** Hana Krskova, Chris Baumann, Yvonne A. Breyer

**Affiliations:** a The Heart Research Institute, Sydney, Australia; b With Focus Consulting, Sydney, Australia; c Macquarie Business School, Macquarie University, Sydney, Australia; d Seoul National University (SNU), Seoul, South Korea, Korea and; e Osaka University, Osaka, Japan

**Keywords:** School discipline, Personal discipline, Parental expectations, Extracurricular activities, Individual competitiveness, Sport, Music

## Abstract

**Purpose:**

This research seeks to extend previous research into student discipline and competitiveness, at the macro or national level, to the micro or individual level. The role of factors with the potential to impact individual competitiveness, namely the expectations of the mother and the father, the importance of school discipline played in primary and secondary schools, and past participation in sport and music were examined.

**Design/methodology/approach:**

Data from a sample of current university students and recent graduates representing Western (the United States) and Asian regions (South Korea and China) were analysed using multiple regressions to establish the explanatory power of independent variables in the competitiveness model, including testing for moderation effects of country of birth.

**Results:**

The study found that personal discipline is significantly associated with individual competitiveness. The importance placed on discipline in primary schools was found to predict individual competitiveness as were parents’ expectations, yet with nuances in terms of maternal and paternal expectations. Surprisingly, neither participation in music nor discipline at secondary school was found to significantly associate with individual competitiveness. At the same time, the study did find moderating effects of country of birth and the number of years students played sport in predicting competitiveness.

**Originality/value:**

Guided by the Ecological Systems Theory and the notion of the Pygmalion effect, we develop a framework of factors that shape an individual's competitiveness. The results make several theoretical contributions by establishing new drivers of individual competitiveness, and as such illuminating the importance of discipline during schooling and how parental expectations drive performance. Implications for employers, educational institutions, and parents are outlined and directions for further research are offered.

## 1 Introduction

“Competition is a market condition, whereas competitiveness is about the ability to create competitive advantage.” [1], p. 64.

Competitive advantage for a firm is underpinned by the knowledge, capabilities [2] and competitiveness of employees. At the individual level, personal competitiveness or the enjoyment of “demonstrating self-competence, mastery, achievement and self-improvement” [3], p. 892 can not only positively impact achievement in education, and work engagement and, in turn, lead to enhanced organisational outcomes, but it can also foster career success. It is, therefore, unsurprising that the role of “interpersonal competition and the desire to win and be better than others” [4], p. 41 has been explored in many domains, such as business [5], sport [6] and education [7].

There are, in fact, many benefits of high levels of competition, requiring competitiveness, or the ability to compete. These include individuals deriving enjoyment from situations involving skill and competition [8], or competition contributing to increased task effectiveness and the drive to tackle increasingly more difficult tasks and projects [9]. Being competitive motivates individual achievement [10] and may also provide a means of improving competence [3]. Furthermore, individual competitiveness incentivises individuals “to outperform others – or at least better one's own performance” [7], p. 189. Last but not least, being competitive propels an individual to aim for success [11].

The purpose of this study was to probe factors with the potential to form an individual's competitiveness. We are driven by a desire to help individuals to enhance their levels of competitiveness to flourish in their choice of personal endeavours, enhance their own employment prospects, as well as contribute – in one way or another - to the creation of a competitive advantage of their employing organisation. One potential driver of competitiveness is discipline, previously found in the context of schools to drive competitiveness at the national level [12]. Other factors linked to greater learning and achievement at school include high expectations from teachers and parents [13] as well as participation in extracurricular activities such as sport or music during school years [14].

What remains underexplored is how discipline, parental expectations and past participation in sport or music - when examined together - impact individual competitiveness. While these drivers have been examined *separately* in the literature, we have extended the investigation into individual competitiveness by *combining* these factors into one comprehensive model to explore relative effects.

The literature points to an interaction effect of the variables under examination in our study with country of birth. For example, notable geographical differences in educational achievements as well as in the levels of discipline have been highlighted by the triennial Programme of International Student Assessment (PISA) [e.g. 15, 16, 17]. Could it be that the cultural background of an individual – we use country of birth as a proxy in our study – has a role to play in the formation of individual competitiveness?

Answering the call for exploration of factors impacting student success not only ‘from a Western perspective’ [18, p. 392] as well as the call to explore vocational behaviour both from Western and Asian perspectives [e.g. [2]], our study was designed to probe drivers of individual competitiveness in both Western and Asian settings. More specifically, our study has been structured to reveal, via a survey instrument, potential variation across three countries, namely South Korea (Korea from here onwards), China and the United States, countries often examined in cross-country studies to compare and contrast culturally disparate achievements in education [e.g. 19, 20, 21].

The specific research questions guiding our investigation are.

(i) Do background factors – such as discipline, parental expectations, and participation in sport or music – predict individual competitiveness?
(ii) Is cultural background a moderator in the formation of individual competitiveness?

The level of competitiveness each individual displays or “the extent to which an individual may feel his or her performance is enhanced by competition” [[3], p. 890] also positively influences organisational outcomes. Gaining a greater knowledge of the drivers of individual competitiveness, therefore, also has implications for employers and managers in the workplace. Such knowledge can not only positively impact the selection process of new staff, but it can also assist with matching individuals to different organisational environments, and may indeed provide input for hiring, and promotion criteria and assessment. This, in turn, can motivate employees to work on increasingly more challenging projects as time passes [9]. In sum, an enhanced understanding of competitiveness can assist employees in thriving at work in so many ways [22], and also benefit their own career advancement and personal fulfilment.

## 2 Literature review

The key concepts that frame this research are individual competitiveness and parental expectations. Additional constructs under investigation include the degree of importance discipline played during primary and secondary education as well as personal discipline; involvement in extracurricular school activities such as in sport and music; and country of birth. The literature review was, therefore, focused on these areas.

### 2.1 Individual competitiveness

At the macro level, competitiveness at national level is something countries strive towards [e.g. [23]], with education found to be a driver for this [24]. Some research has been undertaken to examine competitiveness at the individual level - or ‘micro level’ in economics terminology - as the more competitive individuals are, presumably the higher the competitiveness of the organisation they work for. For instance, Lyons [25] found a strong relationship between the individual competitiveness of sales representatives and the efforts they expended on reshaping their jobs in order to improve sales. In the context of education, Cassidy [26], in a study of 144 undergraduate students, revealed a statistically significant, albeit weak, association between levels of student competitiveness and their preferred approach to learning aimed at higher achievement.

Competitiveness has the potential to positively impact individuals when used towards personal development or improving one's skills, and it has also been found to be linked with optimal psychological health [27]. It is, however, important to note that past research has uncovered that “competitiveness may hold different meanings for people from individualistic and collectivistic cultures” [[28], p. 446]. In fact, it has been noted that “cultural context may be an important moderator of the effect of competition” [29, p. 167]. At the risk of (over-)simplification, individualistic societies place greater value on the needs, goals, preferences and achievements of individuals, while collectivistic societies put family and group goals ahead of the needs of individuals [30]. To explore the moderating effects of country of birth, we tested such factors as moderating effects to explore whether there might be at least some differences between individuals from countries high on individualistic orientation, such as the United States, different to individuals from countries with traditionally collectivistic orientation, such as China and Korea [30].

### 2.2 Parental expectations

The second factor under examination in our study, ‘expectations from parents’, may well have long-term effects for when children grow up and become active in the workforce as adults. The notion that “when we expect certain behaviours of others, we are likely to act in ways that make the expected behaviour more likely to occur” [31, p. 36] underpins the Pygmalion theory; also known as the ‘Rosenthal effect’ [32,33]. The theory posits that high expectations increase achievement, with the positive role of high expectations explored in many areas such as in education [e.g. 34] and in the area of leadership and employee behaviour [e.g. 35]. High expectations have been further popularised in relation to high-performing groups [36]; and the positive role expectations have for teaching in the Fourth Industrial Revolution [37] and beyond.

In the context of education, teachers’ expectations have long been discussed with, for example, Allen [38] highlighting the positive impact of high expectations to use school hours “for solid work instead of being frittered away in play and tittering”, and such high expectations are often coupled with the application of discipline, as “essential to the spirit of study and learning” [38 p. 373]. In relation to the positive impact of parental expectations on student performance, as early as in the nineteen fifties, Campbell [39] discussed the positive impact of home environment, such as the values and attitudes upheld by parents, on school achievement; and the Plowden Report [40], titled *Children and their Primary School*, noted that parental attitudinal factors accounted for almost 60 per cent of the variance in student achievement. In particular, children witnessing no desire in their parents to become involved in school activities are unlikely to develop and internalise positive feelings towards their school [41]. In addition, the importance of discipline in the context of parental expectation was explored, for example, by Shimbo and Tendo [42] in their study of parental pedagogy in Tokyo.

While parental expectations tend to be discussed in the literature as a combined construct, we examine parental expectations (to achieve high marks academically) as two separate, gender-divided constructs – the expectations of the mother and of the father, constituting a novel contribution to the literature. Our study is also novel in our approach not to probe the well-established parental influence on educational performance, but indeed to probe the not yet established effect of parental expectations on competitiveness of an individual.

Hypothesis 1 Mother's expectations of high achievement are significantly associated with individual competitiveness.

Hypothesis 2 Father's expectations of high achievement are significantly associated with individual competitiveness.

### 2.3 Discipline

Discipline has been a topic much discussed by teachers, parents and the popular press as well as educational leaders [e.g. 43, 44]. Since discipline has been found to associate with performance [e.g. 17, 45], we included two types of discipline as predictors of competitiveness, our dependent variable. On the one hand, there is discipline in the context of school, where it is often associated with behaviour management and is applied *externally* by teaching or administrative staff, for instance, by sending a misbehaving pupil to detention. In contrast, as individuals enter their university studies, they are expected to discipline themselves from within to help them achieve better educational outcomes [46]. We refer to the second type of discipline in this study as “personal discipline” in accord with the term “*internal* discipline” [46, p. 47], a skill that can be learnt to assist with “learning, personal development and overall human betterment” [17, p. 1021].

#### 2.3.1 External discipline in education

Discipline has received considerable attention at the school level [e.g. 43, 48], with various attempts made to define and measure the construct [e.g. 49, 50], and with numerous interpretations of discipline developed [e.g. 51, 52]. The advent of a degree of consistency in measuring discipline at school level came about in 2000, when questions about ‘discipline climate’ – measuring student perceptions of discipline – were added to PISA. Run by the OECD three-yearly in nearly 90 countries, PISA is designed to assess academic performance at high school level in the areas of reading, mathematics and science. With the data highlighting a strong association between disciplinary climate and student performance [e.g. 53], studies based on linking PISA data on discipline to academic achievement continue to grow in number [e.g. 17, 54, 55]. Given the established links between school climate and non-cognitive skills [e.g. 56], we hypothesise that the degree of importance discipline played in their schools (both primary and secondary) would have impacted on levels of competitiveness of the individuals we sampled.

Hypothesis 3 The importance of discipline in primary education is significantly associated with individual competitiveness.

Hypothesis 4 The importance of discipline in secondary education is significantly associated with individual competitiveness.

#### 2.3.2 Personal discipline

In the realm of adults, such as in the higher education sector or indeed in the workplace, discipline has been researched far less, with early studies probing discipline being situated – similar to its role at the school level - mostly in the domain of potential drivers of academic performance. For example, Robbins et al. [57] used it to successfully test whether discipline is predictive of the academic performance of students in colleges; and Mattern et al. [58] found that in the context of college admissions process, female students reported higher levels of academic discipline.

When it comes to discipline in the workplace, discipline has been highlighted as essential for continuous improvement [59] and it has also been noted that “creative organizations have discipline at the heart of what they do” [60, p. 206]. In fact, in relation to personal discipline more specifically, our study is aligned with a notion that discipline “is key to success both in education and work” [61, p. 271].

Hypothesis 5 Personal discipline is significantly associated with individual competitiveness.

### 2.4 Involvement in extracurricular school activities

Participation in music and sport might be good examples of when a healthy dose of competitiveness could lead to outstanding results. Interest in the influence of activities such as sport and music has certainly increased following a study into American adolescents’ time use in the late 1980s and early 1990s, derived from a number of large-scale databases, such as the *National Education Longitudinal Study* and the *Longitudinal Study of American Youth of 1988* [62]. Zill and his colleagues found that high school students in year 10 who spent no time on such activities were “57 percent more likely to have dropped out by the time they would have been seniors” in comparison to students who engaged in these activities for between one to 4 h per week [[62], p. 52]. Since then, the positive role of sport and music in the development of individuals has been well supported in the literature [e.g. 14, 19]. Intriguingly, according to a comprehensive review of studies from 1988 to 2003 into extracurricular activities, Feldman and Matjasko [63] uncovered a pattern of school-based activities having a mostly positive effect on adolescent development, as well as a pattern of sport being examined more often than music. Our study design includes both aspects separately, i.e., involvement in sports and music while at school and their impact on competitiveness as an adult.

#### 2.4.1 Involvement in sport

In terms of the link between sport and greater academic achievement, Broh [64, p. 84], for example, revealed in a study of around 25,000 students in eighth grade in the United States that “playing school sports boosts students’ achievement in the classroom and on standardised math tests”. Bradley et al. [65] later reported that participating in school sport provides additional benefits through enhancing conscientiousness and independence, for example, while preparing for Higher Secondary Certificate examinations in the UK. Similarly, in a longitudinal study of 271 high school students in the United States into the relationship between substance use and dropping out of school, Dyer et al. [66] found that participation in sport was positively related to academic achievement. More recently, in a Machine Learning study that focused on the effect of extracurricular activities of 850 bachelor and master students on their academic performance, Rahman et al. [67] reported that students who did not engage in extracurricular activities, such as sport, had lower GPA. These works are the foundation for our sixth hypothesis.

Hypothesis 6 Involvement in sport during school years is significantly associated with individual competitiveness.

#### 2.4.2 Involvement in music

Contrary to the findings of the positive influence of sport on students, less clear-cut findings have been reported from research into the links between academic achievement and studying music or playing an instrument. For example, in a study in the United States comparing math performance or cumulative GPA of high school students, Cox and Stephens [68] found no statistically significant differences between students completing music classes and those not undertaking music classes at school; and in an investigation of the effects of three years of piano instruction on the academic achievement of fourth-grade school pupils, Costa-Giomi [69] uncovered no effect on mathematics or language. In contrast, a study of 4739 elementary and middle school students revealed higher scores in standardised tests in mathematics and English of students participating in music education programs [47]. In an examination of associations between achievement in music and in core academic courses, based on examination data from three consecutive Grade 11 and 12 student cohorts in British Columbia, Canada, Gouzouasis et al. [70] found support for positive links between music participation and higher achievement in mathematics. These associations between participation in music and enhanced academic achievement in school settings would point to the possibility that involvement in music could be pertinent to enhanced learning and academic achievement in the context of higher education.

While past studies predominately focused on the effects of either sport or music individually (or discussed them as part of an assessment of various extracurricular activities [e.g. [14]]), our study was designed to test for the *relative* strength of these two potential predictors together in relation to competitiveness of individuals. In other words, investigating the possible impact that past participation during school years in sport and music activities – the most popular extracurricular activities – might have on the competitiveness of individuals was examined in our research, we believe, for the first time measured separately, and we formulated our seventh hypothesis.

Hypothesis 7 Involvement in music during school years is significantly associated with individual competitiveness.

### 2.5 Country of birth

In many studies on performance, it was established that ‘culture’ in one way or another plays a role, perhaps a moderating role as previously alluded to. Race, country of origin or ethnicity are variables frequently used in cross-cultural research [e.g. 71], as each individual is influenced by the “norms and values of a culture” [72, p. 10]. In our study, we included the variable of ‘country of birth’ for two reasons. Firstly, it is often seen as “a reasonable proxy for cultural differences” [73, p. 657]. Secondly, narrowing down the sample further to respondents born and residing in the three countries under examination was aimed at minimising the impact of “intra-national diversity” [74]. Given the importance of in-country regional and cultural variations in the United States, where a considerable proportion of the population is made up of new migrants, as opposed to Korea that has low incoming migration, selecting ‘country of birth’ reduced that variability.

In relation to the two representatives of the ‘Confucian Orbit’ in our study, Korea and China consistently appear at the top of the academic performance results, as well as reporting high levels of discipline among students, as part of the PISA assessment of academic performance at high school discussed earlier [e.g. 55, p. 55]. Together with the United States, Korea and China also appear to rate well for other factors contributing to competitiveness at a national level [75]: for example, the *World Intellectual Property Organisation,* reporting on the Intellectual Property (IP) filing activity in 2018 [76], listed China first, followed by the United States in second place, with Korea ranked fifth. Taken together, the three countries included in our investigation appear to make a solid foundation for us to compare the three distinct cultures, or more specifically, test ‘country of birth’ as a moderator in the formation of competitiveness.

In terms of our hypothesis that country of birth might influence the relationships between the constructs in our research, examples of cross-cultural research into the competitiveness of students include a study that uncovered a preference of American students towards cooperation and Chinese students for competition as success strategy [77]. In a study of undergraduate students from Australia, Denmark, Hong Kong and Korea, Baumann and Harvey [7] highlighted that individual “competitiveness was significantly and positively associated with academic performance” (p. 196) when probing for moderation effects in relation to the cultural background of the study's respondents. We too were interested in exploring which other factors might explain student competitiveness whilst probing for the potential moderation effect of the country of birth, and this notion was the trigger for our eighth and final hypothesis.

Hypothesis 8 Country of birth is a moderator in the Individual Competitiveness model.

## 3 Conceptual framework and hypotheses overview

**Fig. 1:**
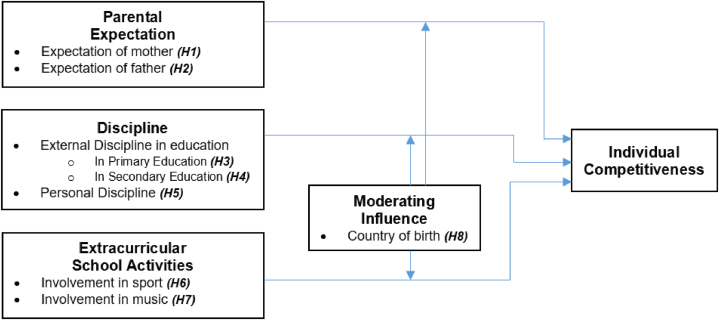
The formation of individual competitiveness: a conceptual framework*.

Source: Authors own development based on the literature review.

Informed by the literature review, we have developed a conceptual model (as detailed in Fig. 1) when theorising about the hypothesised associations of our study. The prime focus of our paper is on the competitiveness of individuals as the ultimate outcome or dependent variable. The independent variables in our study included discipline (both discipline experienced in schools and personal discipline of the respondents), parental expectations, and involvement in sport and music, with country of birth hypothesised to moderate the relationships between these constructs.

Our interest in individual competitiveness was underpinned by the Ecological Systems Theory, which offered a framework for interpreting “the evolving interactions” [78, p. 3] between human beings and their social environment, including the influences of parents and family, school and culture. Furthermore, in the context of the Pygmalion Theory [32,33]), our study explores the impact of high parental expectations on individual competitiveness.

The proposed conceptual model assists with furthering our understanding of the effects of various factors on shaping individual competitiveness. Shedding light on how parental guidance and the values an individual learns at school, through extracurricular activities such as sport and music are linked, or differ, across countries is not only crucial for the field of educational research, but also for the field of, for example, vocational behaviour, particularly when individuals are facing additional impediments to career development as a result of the negative impact of the COVID-19 pandemic [79] or other unexpected circumstances.

Note: *An overview of the theoretical foundation references underpinning this conceptual framework is provided in Table 1.

**Table 1:** Overview of hypotheses with references to prior research.

Hypotheses	Theoretical foundational References
*H1: Mother's expectations of high achievement are significantly associated with individual competitiveness.* *H2: Father's expectations of high achievement are significantly associated with individual competitiveness.* *H3: The importance of discipline in primary education is significantly associated with individual competitiveness.* *H4: The importance of discipline in secondary education is significantly associated with individual competitiveness.* *H5: Personal discipline is significantly associated with individual competitiveness.* *H6: Involvement in sport during school years is significantly associated with individual competitiveness.* *H7: Involvement in music during school years is significantly associated with individual competitiveness.* *H8: Country of birth is a moderator in the individual competitiveness model.*	[80] [80] [12,81] [12,81] [12] [82] [82] [77,83]

The hypotheses associated with the proposed model, based on the review of literature discussed in the previous section, are provided in Table 1.

## 4 Method

### 4.1 Development of survey instrument

The survey questions for measuring the competitiveness and discipline constructs were selected from previously validated questionnaires, while the items utilised for capturing the importance of discipline in education, parental expectations and participation in music and sport were adapted from published studies. An overview of the measurement items that were included in the survey can be found in Appendix A Construct Measurements. All questions were assessed by respondents using a 7-point Likert scale, ranging from 1 (strongly disagree) to 7 (strongly agree).

*Competitiveness* The respondents’ tendency to compete was assessed using a modified version of the *Competitiveness Orientation Measure* [3]. The 37-item COM was designed as a unified measure of trait competitiveness, synthesising previous research on personal enhancement, competitive affectivity, general competitiveness, and dominance dimensions. We selected the two most prominent questions each from three dimensions deemed suitable for assessing student levels of competitiveness: general competitiveness, dominant competitiveness, and personal enhancement competitiveness.

*Discipline* Our study utilised a survey developed for measuring personal discipline in the university sector [84], underpinned by theoretical principles with the potential to impact the discipline of university students, such as *self-determination* [85] or *self-efficacy* [86]. Additional questions were asked about the importance that discipline played in both their primary and secondary education: *Discipline was an important aspect in my primary school, Discipline was an important aspect in my secondary school*. These two statements were inspired by a study into differences of perception between teachers and students about what school discipline constitutes [81].

*Parental expectations* The questions designed to measure parental expectations were based on an academic achievement item (*Parents expect me to have excellent academic performance)* from “a scale for measuring parental expectations and living up to parental expectations” and the impact that expectations have on college students [80, p. 582]. However, in our study we probed the expectations of the mother and those of the father *separately,* making a unique contribution to reveal potential gender differences (i.e. maternal vis-à-vis paternal).

*Participation in music and sport* The respondents were asked about the number of years during their school years (between the ages of 5 and 18) they participated in sport and music activities (separate questions). These items were modelled on questions asked during a study of children's perceptions of their parents' involvement in extracurricular activities [82].

### 4.2 Sample and survey administration

The survey items focusing on the exploration of competitiveness were part of a larger study investigating discipline in the context of higher education. In line with the relevant Ethics Approval, the survey was delivered by an online company specialising in market research with the ability to disseminate it to specific target respondents. The instrument was initially utilised in the American market in order to test the clarity of the questions. Upon a translation by professional Korean and Chinese interpreters, it was pre-tested by a bilingual academic from a related field as well as by a bilingual graduate from an Australian university. This was to ensure the intended meaning of measurement items (such as discipline being an internal mechanism propelling students forward towards their academic goals) was retained.

The objective was to survey recent graduates and current university students and data were collected from a total of 537 respondents from China, Korea and the United States. This exceeds the often cited *N* > 50 + 8 *m* formula for sample size calculations for multiple regression testing, where *m* equals the number of independent variables [87, p. 123]. An overview of the respondents is provided in Appendix B.

### 4.3 Analysis

Multiple linear regression analysis, using IBM SPSS Software, was deemed appropriate for analysing the data in this study because it tests the relationships between one outcome variable and one or more predictors [88]. We were specifically interested in shedding light on the explanatory power the independent variables (IVs) could yield for the dependent variable in our model. Stepwise multiple regression was utilised as it allows optimisation to reveal the order in which the variables are entered into the equation [89] and “to eliminate those IVs that do not provide additional prediction” [87, p. 140]; our aim was to arrive at the most parsimonious model. Prior to interpreting the results of our regression analyses, the data set was assessed for normality and outliers as well as multicollinearity [88] and the tolerance statistics were well above the standard criteria [e.g. 90].

In addition to “demonstrating the existence of an effect” [[91], p. vii] between the variables, we also probed for moderation (interaction effects) of the combined effect of two variables on a third variable [92]. In our analysis, we took the United States as a base and created two indicator variables for Korea and China. Our focus was to reveal possible interaction effects, for example, for the sport variable in combination with country of birth on individual competitiveness (namely: sport * Korea; sport * China).

## 5 Results

Results in relation to the explanatory power of the independent variables under examination on competitiveness are listed first, with the results of the hypothesised interaction effect of a moderating variable on the relationship with individual competitiveness detailed in the following section.

### 5.1 Discipline, parental expectations and involvement in sport explaining competitiveness

We explored whether the associations between the constructs under investigation and individual competitiveness were significant to ascertain the explanatory powers of discipline in primary and secondary education; respondents’ personal discipline; the expectations of the mother and the father of high achievement; and past participation in sport and music. The results of the stepwise regression are summarised in Table 2a. Overall, the explanatory power of the Individual Competitiveness model is 47.1 % (Adjusted *R*^*2*^ = 0.471), indicating that the amount of variance in individual competitiveness explained by our predictors is substantial [88].

**Table:** Table 2bDiscipline, expectations and participation in sport explaining competitiveness – father expectations excluded from regression

Model Standardised coefficients (β) *t* Sig.
(Constant) 2.042 0.042 Personal discipline 0.459 12.319 < 0.001 Expectations mother 0.207 5.450 < 0.001 Importance of discipline in primary school 0.128 3.445 0.001 Sport (in years) 0.080 2.431 0.015 Notes*: n =* 537*, R* = 0.671*, R*^*2*^ = 0.450, Adjusted *R*^*2*^ = 0.445*,* SE of the estimate = 0.9355

**Table 2a:** Discipline, expectations and participation in sport explaining competitiveness – main effects.

Model	Standardised coefficients (β)	*t*	Sig.
(Constant)		2.126	0.034
Personal discipline	0.451	12.554	<0.001
Expectations father	0.275	7.539	<0.001
Sport (in years)	0.088	2.750	0.006
Importance of discipline in primary school	0.098	2.701	0.007

**Notes**.

1) *n = *537*, R* = 0.689*, R*^*2*^ = 0.475, Adjusted *R*^*2*^* = *0.471, SE of the estimate = 0.9137.

2) Expectations of both mothers and fathers were included in the regression, with the expectations of mothers not found to be significant.

**Table 2b:** Discipline, expectations and participation in sport explaining competitiveness – father expectations excluded from regression.

Model	Standardised coefficients (β)	*t*	Sig.
(Constant)		2.042	0.042
Personal discipline	0.459	12.319	<0.001
Expectations mother	0.207	5.450	<0.001
Importance of discipline in primary school	0.128	3.445	0.001
Sport (in years)	0.080	2.431	0.015

**Notes**: n = 537, R = 0.671, R^2^ = 0.450, Adjusted R^2^ = 0.445, SE of the estimate = 0.9355.

Out of the variables under examination in our study, personal discipline was found to have the strongest effect on individual competitiveness (β: 0.451, *p* < 0.001). The results in relation to the role that discipline in schools played were notable in that only the degree to which discipline played a role in primary school was found to significantly impact competitiveness (β: 0.098, *p* = 0.007). In contrast, the effect of discipline in secondary schools was not found to be significant in our model, which will warrant further probe in future research.

The second biggest effect within our regression was the expectations that fathers place on their children in terms of their academic achievement (β: 0.275, *p* < 0.001). When both the expectations of the mother and those of the father were entered into the stepwise regression, the expectations of mothers were not found to significantly impact on competitiveness, suggesting a stronger effect of paternal expectations vis-à-vis maternal.

The results presented in Table 2a beg a question: *what happens when no fatherly expectations of academic achievement are forthcoming in the life of a student?* To see how the explanatory power of our model changes when no father expectations are included in the model (as a simulation of single-mother household settings), as previously alluded to, we re-ran the regressions minus father expectations. The outcome of the second regression analysis is presented in Table 2b.

The explanatory power of the model remained strong at 45.0 % (Adjusted *R*^*2*^ = 0.445), with personal discipline again having the strongest effect (β: 0.451, *p* < 0.001). In relation to the mother's role in shaping competitiveness (such as in single-mother family settings), our simulation revealed that when no fathers' expectations are included in the analysis, mothers' expectations have the second strongest effect (β: 0.207, *p* < 0.001). Our results further revealed that when father expectations are excluded from the study, the students absorb more discipline-related values in the school settings (β: 0.128, *p* = 0.001).

**Fig. 2:**
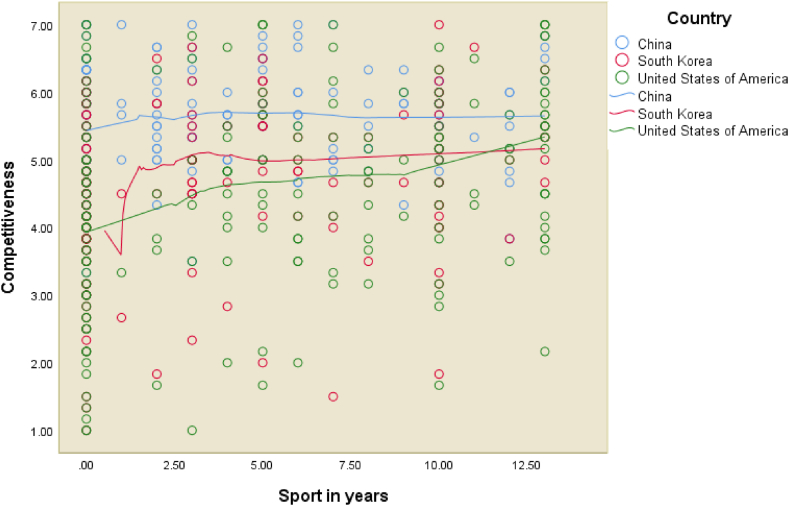
Smooth fitted lines (Loess fit) for competitiveness vs years of sport by country.

In terms of participation in sport and music, the length of time an individual spent playing sport between the age of 5 and 18 was also found to positively impact competitiveness (β: 0.088, *p* = 0.006). The graphical representation of the relationship between years of playing sport and competitiveness is provided in Fig. 2. Overall, the relationship is strongest in China. The respondents from the United States became more competitive with the more years they participated in sport, in stark contrast to those from Korea, who reached their competitive peak after only a few short years of playing sport. The findings relating to music are also noteworthy. While sport impacts the competitiveness of individuals, in our study, music was not found to have a significant effect, warranting future research in this sphere.

### 5.2 The moderating effect of country of birth

**Fig. 3:**
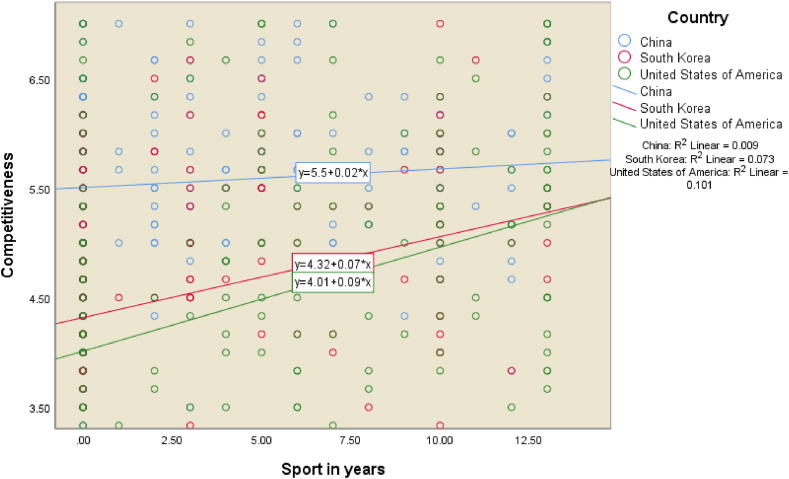
Interaction effects of country of birth and sport on competitiveness.

The second objective of our study was to test for interaction effects in our individual competitiveness model. Out of the variables in our model, only one interaction effect (i.e., country of birth with past participation in sport) was found to be significantly associated with competitiveness. In other words, our analysis revealed that the effect of years of playing sport (between the ages of 5 and 18) on competitiveness is indeed moderated by country of birth (as summarised in Table 3 and depicted in Fig. 2, Fig. 3), but no other moderating effects were found.

**Table 3:** Interaction effects of country of birth in Individual Competitiveness model.

Model	Standardised coefficients (β)	*t*	Sig.
(Constant)		1.270	0.205
Discipline	0.446	12.703	<0.001
Expectations father	0.227	6.247	<0.001
Sport (in years)	0.214	4.468	<0.001
Importance of discipline in primary school	0.071	2.021	<0.044
Korea	0.198	4.107	<0.001
China	0.331	6.425	<0.001
Sport x Korea	−0.065	−1.377	0.169
*Sport x China*	*−0.125*	*−2.539*	*0.011*

**Notes**: n = 537, R = 0.721, R^2^ = 0.520, Adjusted R = 0.513, SE of the estimate = 0.8766.

To illuminate the relationship between years of playing sport and competitiveness, Fig. 2 presents the outcome of a scatterplot after applying the *smoothing Loess fit lines* [93], which revealed a ‘dose response’ effect of the number of years playing sport on competitiveness (as opposed to a ‘threshold’ effect).

The fitted lines show how the relationship in China is at the highest level overall, albeit flatter. More specifically, while the relationship is lower overall, the more years of sport that respondents from the United States played, the more competitive they became. However, for respondents from Korea and China, after playing sport for about two and half years, their competitiveness reached a peak. In China the negative beta coefficient indicates that for every 1-unit increase in the predictor variable, the competitiveness variable will decrease by the value of the beta coefficient (−0.125), with the slope of the line for China being statistically different to the slope of the base American group (Fig. 3).

To recap all the findings of the study in relation to each hypothesis developed on the basis of our review of the literature, Table 4 shows that six of the eight hypotheses are supported.

**Table 4:** Summary of hypotheses.

Competitiveness model hypotheses summary	
*H1: Mother's expectations of high achievement are significantly associated with individual competitiveness.* *H2: Father's expectations of high achievement are significantly associated with individual competitiveness.* *H3: The importance of discipline in primary education is significantly associated with individual competitiveness.* *H4: The importance of discipline in secondary education is significantly associated with individual competitiveness.* *H5: Personal discipline is significantly associated with individual competitiveness.* *H6: Involvement in sport during school years is significantly associated with individual competitiveness.* *H7: Involvements in music during school years is significantly associated with individual competitiveness.* *H8: Country of birth is a moderator in the individual competitiveness model.*	Supported* Supported *Supported* *Not supported* *Supported* *Supported* *Not supported* *Supported*

Note: *When father expectations were excluded from the model.

## 6 Discussion

We applied two lenses to exploring discipline in our study, that is, both the internal role of personal discipline of the respondents, and the role of externally applied discipline in the school context. Our results provide support for our hypothesis that personal discipline is significantly associated with individual competitiveness. Past research has established the link between discipline at the school level and national competitiveness [12], and that the relative impact of discipline on educational performance is higher than the impact of education investment, 88 % and 12 % respectively. Our study now demonstrates that there is also a link between personal discipline and individual competitiveness, this time in the context of higher education.

In terms of the notable difference between the role of discipline in primary school significantly impacting competitiveness but the effect of discipline in secondary schools not been found significant in our model, one explanation for it could be a slow deterioration of interest in schooling (or love of learning) displayed by many school pupils by the time they reach secondary school [94], and/or it could be that school discipline is more pronounced at the elementary vis-à-vis secondary level. While many primary school pupils are ignited by their teachers to explore “almost anything and everything around them”, the secondary school context does not seem to positively influence or contribute to a reversal of their apparent loss of “love of learning” [94, p. 14]. Fortunately, “all humans are born with a hunger to learn, a seemingly insatiable appetite for knowledge” [95, p. 1] and more than half of the approximately 12,000 h students spend in schools is spent in primary schools where pupils eagerly interact and observe teachers, learning by watching while being disciplined by teachers [96]. This could suggest that primary teachers shape and influence children's lives [97], not only through teaching and educating, but also through the importance they place on discipline in primary schools, and indeed as our study now demonstrates, primary school teachers may have a bigger impact on students longer term than high school teachers, at least when it comes to the formation of competitiveness.

In relation to the role parents play in the lives of their offspring, it has been acknowledged that children, and especially children's education, are “dramatically impacted by family structure” [98, p. 149]. When both parents are involved, the role fathers play in the lives of their children in positively influencing individual competitiveness appears to be an area where fathers may be more important than mothers. Our results accords with a study of 1334 families with children aged between five and twelve in the United States where the involvement of fathers was established to affect achievement of students beyond the influence of mothers [98].

Given that 27 % of children in the United States in 2000 lived in single-parent families [99], with the percentage rising to 30 % by 2020 [100], we also explored the scenarios of when fathers might be absent in the family structure, as the effect of the absence of a father in the lives of children is a well-documented topic [e.g. [101]]. Notably, findings of studies into academic achievement of adolescents living in single-father or single-mother households have been inconsistent, with Lee et al. [102, p. 152] reporting “no significant differences in academic achievement” between the two groups. In contrast, Featherman and Hauser [103] reported that children who lived in single-mother families achieved higher academic scores, while Mulkey et al. [104] reported that the absence of a mother was more detrimental to the outcome of science test scores. Our results, when no fathers' expectations were included in the analysis to simulate situations when no fatherly expectations of academic achievement are forthcoming in the life of a student, add to the discussion in the literature. They are in line with Hill and won Kim [105, p. 12] who, in a meta-analysis of 52 studies across 390 correlations between the involvement of fathers and mothers and the academic achievement of their children up to 12th grade, highlighted the positive link between mothers’ involvement and academic achievement.

In regard to sport, our study adds to the discussion of the role of sport in the lives of individuals, particularly around the effects on individual competitiveness, in the context of the moderating effect of country of birth. When it comes to American respondents, the more years of sport the respondents played, the more competitive they became. One of the explanations for sport contributing to enhanced competitiveness (as per Fig. 2) might be because sporting excellence can facilitate entry to university by way of scholarships [106]. Our finding builds on the notion that competitiveness is the essence of sport [107].

While sport impacts the competitiveness of individuals, music was not found to have a significant effect. It appears that while studying music assists, for example, with the development of fine motor skills [108], it does not contribute towards the competitiveness of an individual. One explanation for this finding might rest with the notion that a musical instrument is performed individually, with the player aiming at mastery of a skill [109], while extra-curricular sport activities are predominantly based on groups of participants (about to face another group of players), thus spurring competitiveness on behalf of a team when facing competition.

In sum, our study has provided support for positive responses to the two research questions we posited for this investigation. Specifically.

(iii) We have illuminated the role-specific factors – such as discipline (both in the context of schools and personal discipline), high expectations of parents or participation in sport - play in forming individual competitiveness.
(iv) Furthermore, we have demonstrated that country of birth acts as a moderator in our individual competitiveness model.

## 7 Implications

### 7.1 Theoretical implications

The conceptual model proposed in this study (Fig. 1) provides a new conceptual framework for exploring the links between the influential impact that parents, experiences at school and society in general have on the shaping of individual competitiveness. Adopting a novel perspective of exploring the relationship between the constructs of interest and individual competitiveness, this study was structured to contribute to the body of knowledge by advancing the theoretical understanding of factors impacting individual competitiveness by surveying participants in a university context.

Our study was underpinned by a number of key theories, and it thus makes several noteworthy contributions to the existing theory in several fields of research. We explored individual competitiveness guided by the Ecological Systems Theory, which underscores the influence of social environment, including parents, school and culture [78]. The influence of parental expectations on shaping individual competitiveness is in accord with the Pygmalion Theory [32,33]), which illuminates the impact of high expectations on the life of individuals.

Our study also extends the existing theory on competition. Building on past research that focused on enhancing the theoretical knowledge of variables underpinning constructive organisational outcomes from both Western and Asian perspectives [e.g. [2]], we add to the understanding of determinants of individual competitiveness by exploring both Western and Asian settings, with clear evidence that indeed there are ‘differences’.

As hypothesised, the positive role of parental expectations, external discipline enforced in primary schools as well as personal discipline, together with involvement in sport, for increasing individual competitiveness were supported. We have thus contributed to a better understanding of individual competitiveness by empirically verifying that these four constructs are significantly associated with competitiveness. In contrast, the role of music did not explain individual competitiveness. As such, our results extend the current literature on several topics, such as the significant role that discipline, parental expectations and participation in sport play in the lives of individuals.

### 7.2 Implications for practice

Given the many benefits outlined at the beginning of our paper, in addition to the earlier mentioned theoretical implications, our findings also have important implications for managers and leaders and intriguing implications for educators and parents.

#### 7.2.1 Implications for employers

Based on the links between high levels of individual competitiveness and enhanced performance, as well as the links between competitive advantage and constructive organisational outcomes, organizations could benefit whenever their managers and leaders cultivate individual competitiveness. Enhanced individual competitiveness of employees will, in turn, translate into an enhanced competitive advantage for the employer.

Among some of the reported benefits of competitiveness in the workplace are increased task effectiveness, enhanced enjoyment of work tasks and “motivation to take on challenging projects in the future” [[9], p. 75]. In a marketplace impacted by the flow-on effects of COVID-19, where changing jobs due to dissatisfaction with working conditions is increasingly common, employers might thus consider structuring working environments carefully to harness the benefits of employee competitiveness levels and entice employees into not quitting their jobs due to enjoyment of just the right level of internal (organisational) competition. In other words, employers can benefit from a match between employee's expectations, abilities and skills and their work environment, particularly within a competitive organisational climate.

Employers can also positively influence at least one of the significant constructs in this study: the levels of discipline their staff possess. From a practical perspective, given that personal discipline was found to positively impact individual competitiveness, employers might consider an additional training option in their companies. With discipline being a teachable skill [61], employers could provide support to their employees through implementing discipline elements into their staff development schedules.

#### 7.2.2 Implications for educational institutions

Our study has implications not only in relation to working adults but also to pupils and students as educational institutions are in a position to support individuals in a number of ways. Firstly, they are able to assist students with becoming more disciplined (which in turns positively impacts competitiveness). Similar to the recommendations discussed above in the context of employers, educators could consider incorporating the elements of the skill of discipline into the classroom.

Discipline is not about what not to do, but about what to do [110] and given that personal discipline has been highlighted for having a positive impact in the context of achieving academic excellence [e.g. [111]], students might thus appreciate guidance around how to effectively eliminate distractions when attempting to study. They need to master managing the many competing demands of their studies, particularly if they are enrolled in several subjects. Setting up a schedule – perhaps for each day of the semester– accompanied by when the specific goals (such as assignments) fall due might enable students to take responsibility for being on time with their work, establishing routines and learning to allocate sufficient time to what they are trying to achieve.

Secondly, in relation to competitiveness *per se,* as previously suggested by Baumann and Harvey [7, p. 197], “educators can incorporate an element of competitiveness into the classroom” in the form of, for example, “quizzes, prizes, games, online and physical badges”. Additionally, school career counsellors – after gaining an understanding of a student's individual level of competitiveness – might guide adolescents towards vocational choices that can achieve the closest match between an individual's expectations, abilities and skills and the competitive climate of future workplaces.

#### 7.2.3 Implications for parents

Despite so much emphasis being placed on the contribution of educational institutions to shaping the work readiness of new entrants into the workforce, our study highlights the important role that mothers and fathers play in building the foundations for competitiveness of our future generations. Our results revealed that employers and educators could mainly only assist with one element examined in our study: the enhancement of discipline in individuals to help shape their individual competitiveness. In contrast, and in accord with the notion that parents have a large effect on “many dimensions of their children's lives” [112, p. 21], as well as a recent study that highlighted “the crucial role of parents” in mediating digital media use [113], our results suggest that parents are in a position to enhance their offspring's competitiveness across several of the constructs that our study explored.

Parents could, therefore, consider the levels of expectation they place on their children. We do not invite unnecessary pressure on students from their families; however, our results indicate that high expectations contribute not only to higher academic achievement [e.g. 114, 115] and greater achievement in the workplace [36], but they also positively shape the competitiveness of individuals.

In relation to personal discipline, parents could encourage their children to become more disciplined. By highlighting the importance of focus when children attempt a task and teaching them to eliminate distractions, to allocate enough time while being clear about the goals they are trying to achieve, parents can assist their offspring with gaining a life skill. In addition, parents have been also been acknowledged for playing a key role in their children's sport participation [e.g. 116]. Parents could, therefore, actively encourage their children's participation in sport throughout their primary and secondary schooling.

## 8 Limitations and future research

Research investigations have limitations, and our study is no exception. The potential weakness in our study could be the reliance on self-reported measures, notwithstanding survey studies are the norm in social sciences. Another limitation relates to the retrospective perception of the more mature respondents, who were asked to reflect on their experience in school some years after completing their schooling. At the same time, the very nature of the investigation called for adults to reflect on their upbringing, suggesting that the approach taken was adequate. The spread of the data did not provide cause for concern; there were realistic distributions, indicating that respondents did take the survey seriously and responded to the questions in an honest manner.

Our study was structured to advance the general understanding of individual competitiveness by combining several potential drivers into one model to explore relative importance. There are many factors that impact students’ competitiveness, with the scope of this study having been focused on a specific set of these based on the review of relevant literature. Our results now provide empirical support for theoretical arguments that personal discipline, the level of discipline in primary school, parental expectations of academic achievement, and the number of years of participation in sport between the ages of 5 and 18 indeed contribute to individual competitiveness.

An interesting area for future research would be discipline and parents’ expectations as class-related features: the more affluent are the families, the bigger the expectations and the value of discipline, for instance, enrolling the children in private schools. Hence, further work could test for differences among public, private, selective, and religious schools, to name the most prominent types.^1^

There is, however, scope for more research that would examine the factors of interest in our study; for instance, across more culturally diverse groups within one country and extending the research to more countries, particularly as our analysis revealed that the effect on competitiveness of years of playing sport (between the ages of 5 and 18) is moderated by country of birth. It is, therefore, recommended to explore the associations between sport and individual competitiveness in more detail, for example, by probing for differences between various sports (team vis-à-vis individual sport). Music was not significant in our study, but future research could explore this further, e.g. are there differences based on the type of instrument a child learns, and/or would the type of music studied (i.e. classical or jazz) play a role?

Another line of research could be to examine the individual competitiveness of participants in the workplace across various industries, as well as investigating differences between industries. Given that “Innovation has become the defining challenge for global competitiveness” [117, p. 28], future research could probe potential links between components of a recently introduced M.D.F·C Model of Innovation [118] and individual competitiveness. More specifically, future studies could explore the relationship between individual competitiveness, the skill of discipline, creativity and innovation, and projecting well into the future, the ever-changing role of Artificial Intelligence (AI) could be incorporated into the emerging models on competitiveness. Lastly, multilevel modelling could be considered in future testing since it has been established that issues around competitiveness (e.g. competitive productivity) would interact at three levels (macro, meso and micro) [119], with specific recommendations on the emergent phenomenon and its exploration proposed in recent literature [120].

## 9 Conclusion

The results of this study enhance our understanding of the determinants of individual competitiveness and this paper makes several unique contributions. In the context of the *Ecological Systems Theory,* our study highlights the importance that social environments - including the influence of school, parents and culture - play in shaping individual competitiveness. We present evidence in support of the positive role of upholding the importance of discipline in primary education, and of the number of years that individuals participate in sport between the ages of 5 and 18, in explaining competitiveness. Against the backdrop of the *Pygmalion effect* (or, more specifically, the expectations placed on students by their parents), our study provides evidence of the gender effect of parents in influencing individual competitiveness, particularly around how mother's and father's expectations differ in shaping competitiveness. The study has further established that the moderation effect of country of birth with past participation in sport was significantly associated with competitiveness.

In conclusion, it has been established that regardless of age, people engage “in some competitive activity” almost daily [9, p 63], thus our study has implications for all realms of an individual's life, pretty much at any stage of career and life. Given the teachable nature of the skill of discipline and the significant influence personal discipline plays in the context of individual competitiveness, we recommend that individuals, parents, businesses, and educational providers collectively look for opportunities to enhance the skill of discipline of students and workforce participants alike. This, in turn, will positively impact individual competitiveness. And the higher the levels of individual competitiveness, the more that can be achieved in the competitive marketplace overall, today and into the future.

## Data availability

Data will be made available on reasonable request.

## Ethics Approval details

Institution: Macquarie University, Sydney, Australia.

The full name of the ethics committee: Faculty of Business & Economics - Human Research Ethics Sub Committee.

The approval number: Ref: 5201700956.

Informed consent obtained: Yes, while administering the survey.

## CRediT authorship contribution statement

**Hana Krskova:** Writing – review & editing, Writing – original draft, Visualization, Validation, Software, Project administration, Methodology, Investigation, Funding acquisition, Formal analysis, Data curation, Conceptualization. **Chris Baumann:** Writing – review & editing, Validation, Supervision, Methodology, Conceptualization. **Yvonne A. Breyer:** Writing – review & editing, Supervision, Methodology, Conceptualization.

## Declaration of competing interest

No potential conflict of interest was reported by the authors.

## Figures and Tables

**Table:** 

Construct	Items	References
Personal Discipline	*Sample of F·I.R.S.T. Discipline Instrument items* •I am able to reduce interruptions during the period of time I have set aside for studying •I am able to say “no” to social pressure like going out with friends if I know I have to study •feel enthusiastic about my learning •I have clear goals that I aspire to achieve •I feel in control of what academic results I achieve at university •I use a specific “per day” schedule or timetable to ensure I know what is happening when •I am on time to my classes •I allocate specific amount of time per day to a certain task	[84]
Importance of discipline in primary education	•Discipline was an important aspect in my primary school.	[81]
Importance of discipline in secondary education	•Discipline was an important aspect in my secondary school.	[81]
Expectations mother	•My mother places high expectations on me to achieve high marks academically	[80]
Expectations father	•My father places high expectations on me to achieve high marks academically	[80]
Involvement in sport	•Between the ages 5 and 18, how many years did you play sport for?	[82]
Involvement in music	•Between the ages 5 and 18, how many years did you play music for?	[82]
Competitiveness	•Other people comment on how competitive I am •I try to be the best person in the room at almost anything •Competition motivates me •I perform better when I compete against others •I can improve my competence by competing •Competition allows me to judge my level of competence	[[3], p. 894]

Note 1: Items were measured by utilising a seven-point Likert scale, ranging from 1 (strongly disagree) to 7 (strongly agree).

**Table:** 

China Korea United States Total
Size *N* 172,181,184 537 (%) 32 33.7 34.3 Gender Male (%) 51.7 49.7 48.9 50.1 Female (%) 48.3 50.3 51.1 49.9 Age 18–24 (%) 54.1 35.9 26.1 38.4 24-34 (%) 37.2 28.2 28.8 31.3 35-44 (%) 5.8 23.2 10.3 13.2 Over 45 (%) 2.9 12.7 34.8 17.1

## Footnotes

1 The authors would like to thank an anonymous reviewer for the suggestion.

## References

[1] Baumann C., Hoadley S., Hamin H., Nugraha A. (2017). Competitiveness vis-à-vis service quality as drivers of customer loyalty mediated by perceptions of regulation and stability in steady and volatile markets. J. Retailing Consum. Serv..

[2] Shanker R., Bhanugopan R., Van der Heijden B.I., Farrell M. (2017). Organizational climate for innovation and organizational performance: the mediating effect of innovative work behavior. J. Vocat. Behav..

[3] Newby J.L., Klein R.G. (2014). Competitiveness reconceptualized: psychometric development of the competitiveness orientation measure as a unified measure of trait competitiveness. Psychol. Rec..

[4] Spence J., Helmreich R.L., Spence J.T. (1983). Achievement and Achievement Motives: Psychological and Sociological Approaches.

[5] Lam L.W. (2012). Impact of competitiveness on salespeople's commitment and performance. J. Bus. Res..

[6] Jang W., Kwak D.H., Ko Y.J. (2020). Vitalizing effect of athlete-drafting task in fantasy sports: the role of competitive goal-framing, involvement, and competitiveness trait. Eur. Sport Manag. Q..

[7] Baumann C., Harvey M. (2018). Competitiveness vis-à-vis motivation and personality as drivers of academic performance – introducing the MCP model. Int. J. Educ. Manag..

[8] Spence J., Helmreich R.L. (1978).

[9] Tjosvold D., Johnson D.W., Johnson R.T., Sun H. (2003). Can interpersonal competition be constructive within organizations?. J. Psychol..

[10] Hibbard D.R., Buhrmester D. (2010). Competitiveness, gender, and adjustment among adolescents. Sex. Roles.

[11] Rivalries Simmons M. (2016). insecurities, and dance competition: can you escape the pressure?. Dance Major Journal.

[12] Krskova H., Baumann C. (2017). School discipline, investment, competitiveness and mediating educational performance. Int. J. Educ. Manag..

[13] Rubie-Davies C.M., Peterson E., Irving E., Widdowson D., Dixon R. (2010). Expectations of achievement: student, teacher and parent perceptions. Res. Educ..

[14] Eccles J.S., Barber B.L., Stone M., Hunt J. (2003). Extracurricular activities and adolescent development. J. Soc. Issues.

[15] OECD (2012).

[16] Jerrim J. (2015). Why do East Asian children perform so well in PISA? An investigation of Western-born children of East Asian descent. Oxf. Rev. Educ..

[17] Baumann C., Krskova H. (2016). School discipline, school uniforms and academic performance. Int. J. Educ. Manag..

[18] McInerney D.M., Schunk D.H., Zimmerman B.J. (2012). Motivation and Self-Regulated Learning: Theory, Research, and Applications.

[19] Cheng P.S.-T., Stier Jr WF., Kim C., Xu B.-L., Koshimizu E., Koozechian H. (2004). A comparison of recreational sports and leisure time participation of college/university students in China, Japan, Korea, Iran, the United States, and Canada—with students in the Republic of China (taiwan). Recreat. Sports J..

[20] Galton M., Lai K.C., Chan K.W. (2019). Implementing small class teaching in East Asia: problems and possibilities. Int. J. Educ. Res..

[21] Wang C., Kolano L., Kim D.-H. (2020). Educational Practices in China, Korea, and the United States: Reflections from a Study Abroad Experience.

[22] Babalola M.T., Ren S., Ogbonnaya C., Riisla K., Soetan G.T., Gok K. (2022). Thriving at work but insomniac at home: understanding the relationship between supervisor bottom-line mentality and employee functioning. Hum. Relat..

[23] Schwab K. (2017).

[24] Baumann C., Winzar H. (2016). The role of secondary education in explaining competitiveness. Asia Pac. J. Educ..

[25] Lyons P. (2006). Individual competitiveness and spontaneous changes in jobs. Journal of Competitiveness Studies.

[26] Cassidy S. (2008). Approaches to learning and competitive attitude in students in higher education. The Psychology of Education Review.

[27] Ryckman R.M., Hammer M., Kaczor L.M., Gold J.A. (1996). Construction of a personal development competitive attitude scale. J. Pers. Assess..

[28] King R.B., McInerney D.M., Watkins D.A. (2012). Competitiveness is not that bad at least in the East: testing the hierarchical model of achievement motivation in the Asian setting. Int. J. Intercult. Relat..

[29] Schneider B.H., Woodburn S., del Toro MdPS., Udvari S.J. (2005). Cultural and gender differences in the implications of competition for early adolescent friendship. Merrill-Palmer Q..

[30] Hofstede G., Hofstede G.J., Minkov M. (2010).

[31] Rosenthal R., Babad E.Y. (1985). Pygmalion in the gymnasium. Educ. Leader.

[32] Rosenthal R. (1963). On the social psychology of the psychological experiment: 1, 2 the experimenter's hypothesis as unintended determinant of experimental results. Am. Sci..

[33] Rosenthal R. (1966). Experimenter Effects in Behavior Research. New York: Appleton-Century-Crofts.

[34] Niari M., Manousou E., Lionarakis A. (2016). The pygmalion effect in distance learning: a case study at the Hellenic Open University. Eur. J. Open Dist. E Learn..

[35] Duan J., Li C., Xu Y., Wu Ch (2017). Transformational leadership and employee voice behavior: a Pygmalion mechanism. J. Organ. Behav..

[36] Coyle D. (2018).

[37] Doucet A., Evers J., Guerra E., Lopez N., Soskil M., Timmers K. (2018). Teaching in the Fourth Industrial Revolution: Standing at the Precipice.

[38] Allen F.S. (1918). School discipline. J. Educ..

[39] Campbell W. (1952). The influence of home environment on the educational progress of selective secondary school children. Br. J. Educ. Psychol..

[40] Plowden B. (1967).

[41] Kohl D., Recchia S., Steffgen G. (2013). Measuring school climate: an overview of measurement scales. Educ. Res..

[42] Shimbo A., Tendo M. (2022). Creating cultural resources and reading: a case study of a public library and invisible parental pedagogy in Tokyo. Int. J. Educ. Res..

[43] Slee R. (1988). Discipline and Schools: A Curriculum Perspective.

[44] Pasternak R. (2013). Discipline, learning skills and academic achievement. Journal of Arts and Education.

[45] Romi S., Lewis R., Katz Y.J. (2009). Student responsibility and classroom discipline in Australia, China, and Israel. Compare.

[46] Krskova H., Breyer Y., Baumann C., Wood L.N. (2019). An exploration of university student perceptions of discipline: introducing F.I.R.S.T. Discipline principles. High Educ. Skills Work. base Learn..

[47] Johnson C.M., Memmott J.E. (2006). Examination of relationships between participation in school music programs of differing quality and standardized test results. J. Res. Music Educ..

[48] Millei Z., Griffiths T.G., Parkes R.J. (2010). Re-theorizing Discipline in Education: Problems, Politics, & Possibilities.

[49] Lewis R. (2001). Classroom discipline and student responsibility:: the students' view. Teach. Teach. Educ..

[50] Ferreira A., Jacobs L., De Wet C., Coetzee-Manning D. (2009). Discipline in Lesotho schools: educator strategies. Acta Acad..

[51] Jones F.H. (1987).

[52] Oplatka I., Atias M. (2007). Gendered views of managing discipline in school and classroom. Gend. Educ..

[53] OECD (2011).

[54] Chiu M.M., Chow B.W.Y. (2011). Classroom discipline across forty-one countries: school, economic, and cultural differences. J. Cross Cult. Psychol..

[55] Baumann C., Winzar H., Viengkham D. (2020).

[56] Zynuddin S.N., Kenayathulla H.B., Sumintono B. (2023). The relationship between school climate and students' non-cognitive skills: a systematic literature review. Heliyon.

[57] Robbins S.B., Allen J., Casillas A., Peterson C.H., Le H. (2006). Unraveling the differential effects of motivational and skills, social, and self-management measures from traditional predictors of college outcomes. J. Educ. Psychol..

[58] Mattern K., Sanchez E., Ndum E. (2017). Why do achievement measures underpredict female academic performance?. Educ. Meas..

[59] Senge P.M. (2006).

[60] Napier N.K., Nilsson M. (2008).

[61] Krskova H., Breyer Y., Baumann C., Wood L.N., Breyer Y.A., Tan L.P., Wood L.N. (2020). Industry and Higher Education: Case Studies for a Sustainable Future.

[62] Zill N., Nord C.W., Loomis L.S. (1995).

[63] Feldman A.F., Matjasko J.L. (2005). The role of school-based extracurricular activities in adolescent development: a comprehensive review and future directions. Rev. Educ. Res..

[64] Broh B.A. (2002). Linking extracurricular programming to academic achievement: who benefits and why?. Sociol. Educ..

[65] Bradley J., Keane F., Crawford S. (2013). School sport and academic achievement. J. Sch. Health.

[66] Dyer A.M., Kristjansson A.L., Mann M.J., Smith M.L., Allegrante J.P. (2017). Sport participation and academic achievement: a longitudinal study. Am. J. Health Behav..

[67] Rahman S.R., Islam M.A., Akash P.P., Parvin M., Moon N.N., Nur F.N. (2021). Effects of co-curricular activities on student's academic performance by machine learning. Current Research in Behavioral Sciences.

[68] Cox H., Stephens L. (2006). The effect of music participation on mathematical achievement and overall academic achievement of high school students. Int. J. Math. Educ. Sci. Technol..

[69] Costa-Giomi E. (2004). Effects of three years of piano instruction on children's academic achievement, school performance and self-esteem. Psychol. Music.

[70] Gouzouasis P., Guhn M., Kishor N. (2007). The predictive relationship between achievement and participation in music and achievement in core grade 12 academic subjects. Music Educ. Res..

[71] Glass C.R., Westmont C.M. (2014). Comparative effects of belongingness on the academic success and cross-cultural interactions of domestic and international students. Int. J. Intercult. Relat..

[72] Löhr A., Steinmann H., Lange H., Löhr A., Steinmann H. (1998). Working across Cultures - Ethical Perspectives for Intercultural Management.

[73] McMurray A., Scott D. (2013). Work values ethic, GNP per capita and country of birth relationships. J. Bus. Ethics.

[74] Tung R.L., Baumann C. (2009). Comparing the attitudes toward money, material possessions and savings of overseas Chinese vis-a-vis Chinese in China: convergence, divergence or cross-vergence, vis-a-vis ‘one size fits all’human resource management policies and practices. Int. J. Hum. Resour. Manag..

[75] Schwab K., Sala-i-Martín X. (2020).

[76] WIPO. World Intellectual Property Indicators 2019. Geneva: World Intelectual Proporty Organisation 2019..

[77] Tang S. (1999). Cooperation or competition: a comparison of US and Chinese college students. J. Psychol..

[78] Bronfenbrenner U. (1979).

[79] Spurk D., Straub C. (2020). Flexible employment relationships and careers in times of the COVID-19 pandemic. J. Vocat. Behav..

[80] Wang L.-F., Heppner P.P. (2002). Assessing the impact of parental expectations and psychological distress on Taiwanese college students. Counsel. Psychol..

[81] Haroun R., O'Hanlon C. (1997). Do teachers and students agree in their perception of what school discipline is?. Educ. Rev..

[82] Anderson J.C., Funk J.B., Elliott R., Smith P.H. (2003). Parental support and pressure and children's extracurricular activities: relationships with amount of involvement and affective experience of participation. J. Appl. Dev. Psychol..

[83] Ryckman R.M., Van den Borne H., Syroit J. (1992). Differences in hypercompetitive attitude between American and Dutch university students. J. Soc. Psychol..

[84] Krskova H., Baumann C., Breyer Y., Wood L.N. (2020). The skill of discipline–measuring FIRST discipline principles in higher education. High Educ. Skills Work. base Learn..

[85] Deci E.L., Ryan R. (1985).

[86] Bandura A. (1977). Self-efficacy: towards a unifying theory and the organization. Psychol. Rev..

[87] Tabachnick B.G., Fidell L.S. (2013).

[88] Muijs D. (2011).

[89] Pallant J. (2016).

[90] Hair J.F., Black W.C., Babin B.J., Anderson R.E. (2014).

[91] Hayes A.F. (2018).

[92] Field A. (2013).

[93] Cleveland W.S. (1979). Robust locally weighted regression and smoothing scatterplots. J. Am. Stat. Assoc..

[94] Sarason S.B. (1990).

[95] Lumsden L. (1999).

[96] Curwin R.L., Mendler A.N. (1988).

[97] Christensen C.A., Massey D.R., Isaacs P.J., Synott J. (1995). Beginning teacher education: students' conceptions of teaching and approaches to learning. Australian Journal of Teacher Education.

[98] McBride B.A., Schoppe-Sullivan S.J., Ho M.-H. (2005). The mediating role of fathers' school involvement on student achievement. J. Appl. Dev. Psychol..

[99] Sigle-Rushton W., McLanahan S., Moyniham D., Smeeding T., Rainwaters L. (2004). The Future of the Family.

[100] Hemez P., Washington C. (2021). https://www.census.gov/library/stories/2021/04/number-of-children-living-only-with-their-mothers-has-doubled-in-past-50-years.html.

[101] Barajas M.S. (2011). Academic achievement of children in single parent homes: a critical review. The Hilltop Review.

[102] Lee S.M., Kushner J., Cho S.H. (2007). Effects of parent's gender, child's gender, and parental involvement on the academic achievement of adolescents in single parent families. Sex. Roles.

[103] Featherman D.L., Hauser R.M. (1978).

[104] Mulkey L.M., Crain R.L., Harrington A.J. (1992). One-parent households and achievement: economic and behavioral explanations of a small effect. Sociol. Educ..

[105] Hill N.E., won Kim S. (2015). Including fathers in the picture: a meta-analysis of parental involvement and students' academic achievement. J. Educ. Psychol..

[106] Camiré M. (2014). Youth development in North American high school sport: review and recommendations. Quest.

[107] Yang Z., Zhao G. (2011). Sport and competitiveness. J. Phys. Educ..

[108] Costa‐Giomi E. (2005). Does music instruction improve fine motor abilities?. Ann. N. Y. Acad. Sci..

[109] Gardner H. (2008).

[110] Charles C.M., Barr K.B. (1992).

[111] Halstead J. (2014). Invigorating Classrooms in a Common Core Environment.

[112] Becker G.S. (1993).

[113] Sun H., Lim V., Low J., Kee S. (2022). The development of a parental questionnaire (QQ-MediaSEED) on bilingual children's quantity and quality of digital media use at home. Acta Psychol..

[114] Baumrind D. (1966). Effects of authoritative parental control on child behavior. Child Dev..

[115] Spera C. (2005). A review of the relationship among parenting practices, parenting styles, and adolescent school achievement. Educ. Psychol. Rev..

[116] Bonavolontà V., Cataldi S., Latino F., Carvutto R., De Candia M., Mastrorilli G. (2021). The role of parental involvement in youth sport experience: perceived and desired behavior by male soccer players. Int. J. Environ. Res. Publ. Health.

[117] Porter M., Stern S. (2001). Innovation: location matters. MIT Sloan Manag. Rev..

[118] Krskova H., Breyer Y.A. (2023). The influence of growth mindset, discipline, flow and creativity on innovation: introducing the MDFC model of innovation. Heliyon.

[119] Baumann C., Cherry M., Chu W. (2019). Competitive Productivity (CP) at macro–meso–micro levels. Cross Cult. Strateg. Manag..

[120] Winzar H., Baumann C., Soboleva A., Park S.H., Pitt D. (2022). Competitive Productivity (CP) as an emergent phenomenon: methods for modelling micro, meso, and macro levels. Int. J. Hospit. Manag..

